# Development of Thermoresponsive Composite Hydrogel Loaded with Indocyanine Green and Camptothecin for Photochemotherapy of Skin Cancer After Surgery

**DOI:** 10.3390/gels11010071

**Published:** 2025-01-16

**Authors:** Yu-Hsiang Lee, Chieh-Lin Chung

**Affiliations:** 1 Department of Biomedical Sciences and Engineering, National Central University, Taoyuan City 320317, Taiwan; jlinchung1102@gmail.com; 2 Department of Chemical and Materials Engineering, National Central University, Taoyuan City 320317, Taiwan; 3 Department of Medical Research, Cathay General Hospital, Taipei City 106438, Taiwan

**Keywords:** skin cancer, injectable hydrogel, thermoresponsive, hyaluronic acid, photochemotherapy, near infrared

## Abstract

Skin cancer is the world’s fifth most diagnosed malignancy and is increasingly occurring in young adults. The elevated morbidity and mortality of skin cancer are known to be highly correlated with its frequent recurrence after tumor excision. Although regimens such as chemotherapy and/or immunotherapy are often administered following surgical treatments, the patients may suffer from severe side effects, drug resistance, and/or high cost during treatments, indicating that the development of an effective and safe modality for skin cancer after surgery is still highly demanded nowadays. In this study, an injectable and thermoresponsive hyaluronic acid/hexamethylene diisocyanate-Pluronic F127 block copolymer crosslinking composite hydrogel loaded with indocyanine green (ICG) and camptothecin (CPT), called ICHHPG, was developed for photochemotherapy of skin cancer after surgery. ICHHPG can be self-gelationed at 37 °C and stabilizes ICG in the gel matrix. Upon NIR exposure, ICHHPG can generate hyperthermia and consequently provide photothermal therapy when the ICG dosage is >5 μM. Furthermore, ICHHPG may provide a remarkably enhanced cancericidal effect compared to the equal concentration of free ICG (≤10 μM) or CPT (≤1000 μM) alone, and more than 95% of cancer cells can be destroyed as the intra-gel doses of ICG/CPT were elevated to 10/800 μM. Given the confirmed cytotoxicity together with its fluidic and thermoresponsive characteristics which are foreseeably favorable for wound coverage, the developed ICHHPG is highly applicable for use in skin cancer treatment after surgical excision.

## 1. Introduction

Skin cancer is the fifth most diagnosed neoplastic disease worldwide and is one of the most common cancers in young adults, despite the risk of skin cancer generally increasing with age [1,2]. Skin carcinoma is typically categorized into melanoma skin cancer (MSC, melanocytes) and non-melanoma skin cancer (NMSC, keratinocytes) based on the cellular origin, and is preferably treated with surgical excision (SE) during its early stages [3,4,5]. Although the latter accounts for >90% of all skin cancer cases, the former, with 80% of mortality, causes the majority of skin cancer deaths due to its high metastasis nature [6]. Moreover, the recurrence of malignance plays a fatal role in elevated morbidity and mortality of skin cancer [7]. Although systemic therapeutics such as radiotherapy, chemotherapy, target therapy, and immunotherapy may abolish the recurrence of skin cancer [8,9,10,11], it suffers from severe side effects, drug resistance, poor bioavailability, and/or high cost with the current treatment strategies [12,13,14], suggesting that an effective and safe adjuvant modality for skin cancer post-surgery is still highly desired nowadays.

Local drug delivery directly operated at the surgical/radiotherapeutic sites may be a promising strategy to reduce the aforementioned issues after surgical excision. Hydrogel is an appropriate dressing material because it can provide excellent biocompatibility, biodegradability, hydrophilicity, and similar mechanical properties with the natural extracellular matrix [15]. Moreover, injectable hydrogels which are able to reverse sol–gel phase transition in response to environmental stimuli such as temperature and/or pH [16,17], have gained increasing attention on medical uses because they can adapt the size/shape of the lesion, enable controlled drug release, facilitate wound healing, and reduce the risks of infection and/or complications reported previously [15].

Among various biocompatible molecules used for hydrogel preparation, Pluronic F127 (PF127) is a synthetic temperature-sensitive poly(propylene oxide) (PPO) polymer flanked by poly(ethylene oxide) (PEO) blocks and has been extensively used in a variety of biomedical applications such as cell culture, tissue engineering, topical drug transport etc. [18,19,20]. However, the application of PF127 is dramatically hindered by its weak modulus and rapid disintegration [21], indicating that modification of PF127 for the enhancement of its mechanical properties is highly demanded before utilization.

Joint therapy through the administration of multiple cancericidal agents and/or modalities is a feasible manner to decrease multidrug resistance since the effective dose and/or accumulation of each anticancer drug in tumors can be reduced, and therefore the issue of drug resistance can be solved [22]. Among various chemotherapy adjuvants, near-infrared (NIR)-mediated photothermal therapy (PTT) has long been recognized as a feasible approach because it can provide several advantages such as noninvasiveness, superior tissue penetration efficacy, and enhanced drug transport and/or absorption efficiency [23,24]. In general, PTT works through thermal ablation generated by photosensitizers upon light exposure, whereby irreversible cell damage can be made as the temperature is minimally over 42 °C [25].

Indocyanine green (ICG) is a USFDA-approved photosensitizer and has been widely used in neoplastic PTT including colorectal, breast, and cervical cancers [26,27,28] since it is able to generate hyperthermia upon NIR irradiation. However, drawbacks such as thermal- and photo-susceptibility, concentration-dependent agglomeration, and quick clearance in circulation [29,30] dramatically hinder its application in the clinic.

Hydrogel-mediated drug delivery may provide a feasible method to handle and stabilize multiple agents, such as ICG and chemo-drugs, in the transport system. In this study, we aimed to develop an injectable and thermoresponsive hexamethylene diisocyanate (HDI)-PF127-hyaluronic acid (HA) composite hydrogel bearing ICG and camptothecin (CPT), called ICHHPG, for the photochemotherapy of skin cancer after surgery. CPT is an FDA-approved anticancer drug which can change the structure of Topoisomerase I to inactivate the transcription function of cancer cells, leading to apoptosis accordingly [31]. HA is employed because there is overexpression of HA receptor CD44 on tumor cells, suggesting that HA is suitable for use in anticancer drug delivery [32]. In this paper, the fabrication, characterization, and in vitro anticancer functionalities of ICHHPG are investigated stepwise.

## 2. Results and Discussion

### 2.1. Characterization of ICHHPG

**Figure 1:**
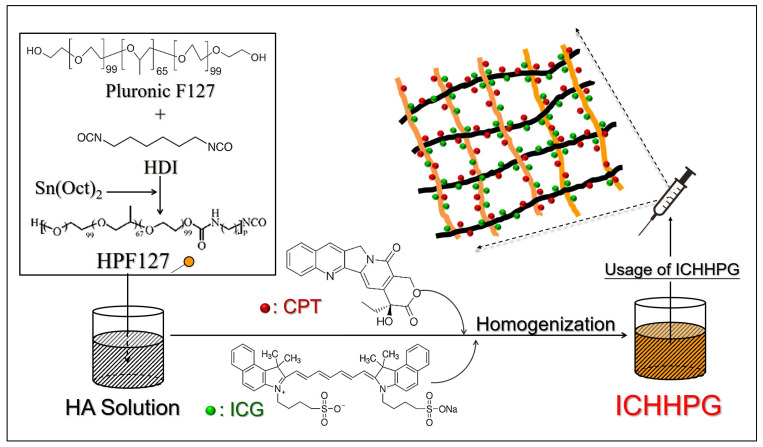
Schematic diagram of ICHHPG fabrication.

**Figure 2:**
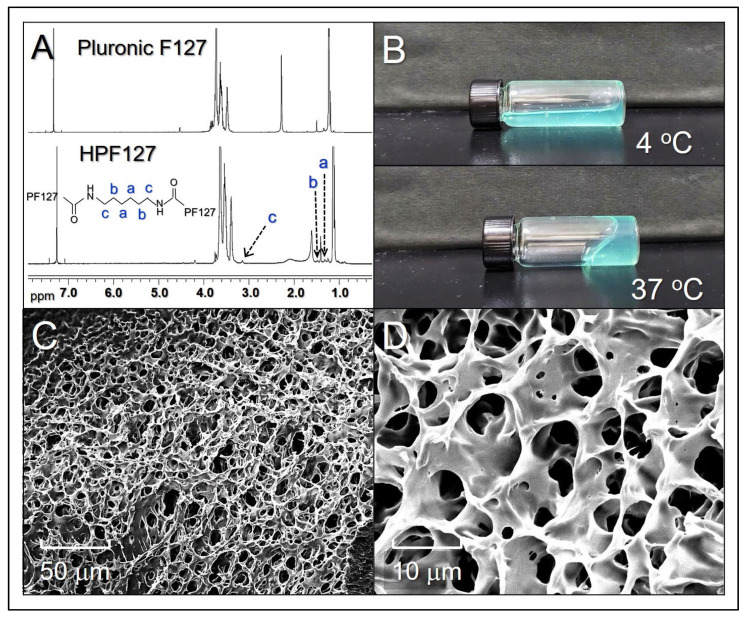
(**A**) The ^1^H NMR spectra of PF127 and the synthesized HPF127. The a, b, and c at 1.3, 1.5, and 3.2 ppm in the HPF127 spectrum represent the methylene protons of aliphatic chain of HDI. (**B**) Photographs of the real ICHHPG samples under 4 or 37 °C. (**C**,**D**) SEM images displaying the surface (**C**) and inner structure (**D**) of ICHHPG at 2000X (**C**) and 10,000X (**D**) magnification, respectively.

The procedures of ICHHPG fabrication are schematically diagrammed in Figure 1. Figure 2A shows the ^1^H NMR spectra of PF127 (Figure 2A, top) and HDI-PF127 (HPF127, Figure 2A, bottom) polymers. It could be observed that both PF127 and HPF127 exhibit ethylene protons of PEO and methyl protons of PPO at 3.64 and 1.05 ppm, respectively, while only the latter shows the methylene protons at 1.3, 1.5, and 3.2 ppm that represent the characteristics of the aliphatic chain of HDI. These results illustrate that the HPF127 was formed with both PF127 and HDI structures, demonstrating that the polymer synthesis was successfully fulfilled in this study.

ICHHPG is a turquoise translucent injectable hydrogel (Figure 2B). Based on its fluidic conditions under different temperatures, ICHHPG exhibited a relatively high fluidity (i.e., lower viscosity) in 4 °C (Figure 2B, top) compared to that under 37 °C (Figure 2B, bottom), illustrating that ICHHPG is performed with thermoresponsive characteristics. Since the critical micelle concentration (CMC) of PF127 is reversely proportional to the environmental temperature [33,34], we speculate that the temperature-sensitivity of ICHHPG is attributed to the micellar self-assembly property of PF127, by which increased amount of HPF127 micelles generated and agglomerated to form a gel when the temperature is elevated, rendering ICHHPG a capability of sol–gel phase transition with temperature variation. Furthermore, ICHHPG is formed with a porous/network structure as shown in Figure 2C,D that is favorable for the intra-gel diffusion of CPT as well as drug release from the gel.

### 2.2. Thermal Stability of ICHHPG-Entrapped ICG

**Figure 3:**
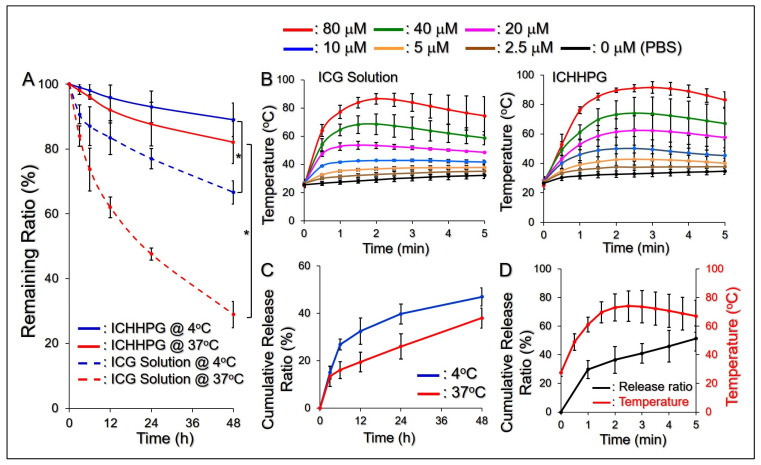
Thermal stability and functionality of ICHHPG in vitro. (**A**) Quantitative analyses of the amount of ICG remaining in DI water or ICHHPG under incubation at 4 or 37 °C within 48 h. * *p* < 0.05. (**B**) Hyperthermia effects of free ICG (left) and ICHHPG (right) under NIR exposure for 5 min. (**C**) CPT release profiles of ICHHPG under 4 or 37 °C within 48 h. (**D**) CPT release profile of ICHHPG under 5 min of NIR irradiation (black curve). The red curve indicates the temperature variation in the system within 5 min NIR exposure. Values in (**A**–**D**) are the mean ± SD (n = 3). * *p* < 0.05.

Figure 3A exhibits the degradation profiles of ICG in different settings under 4 or 37 °C within 48 h. Our data show that ICG in aqueous solution dramatically decayed compared to that in ICHHPG under equal temperature setting (*p* < 0.05 for each). Based on the spectrometric measurement, approximately 90% and 80% of the entrapped ICG can be remained in the hydrogel after incubation at 4 or 37 °C for 48 h, while that of 65% and 30% were maintained in the aqueous solution. These results indicate that ICHHPG is able to improve the thermal stability of the loaded ICG, and we reason that such effect is attributed to the protections by the HHPG.

### 2.3. Effects of Hyperthermia on ICHHPG

Figure 3B exhibits the efficacies of heat production of the ICG solution and ICHHPG with different ICG concentrations within 5 min of NIR exposure. Both groups displayed similar temperature raising patterns and showed dose-dependent hyperthermia effects under NIR irradiation. In addition, the hyperthermia generated by ICHHPG was slightly higher than the ICG solution under the same concentration setting. We surmise that it was because ICHHPG shifted toward the gel phase at a higher temperature (Figure 2B) whereby the kinetics of molecular movement reduced and more thermal energy was preserved in the system, consequently leading to a higher temperature. These outcomes indicate that ICHHPG is certainly able to generate hyperthermia and consequently provide effective PTT (*T* ≥ 42 °C) as the concentration of the loaded ICG is more than 5 μM.

### 2.4. Drug Release Kinetics of ICHHPG

Figure 3C shows drug release profiles of ICHHPG-entrapped CPT under 4 and 37 °C within 48 h. The gel exhibited a two-stage release profile consisting of a rapid release in the first 3 h at 37 °C, or 6 h at 4 °C, followed by a slowly sustained release, giving a cumulative release ratio of approximately 47% at 4 °C, or 38% at 37 °C, after 48 h. The circumstance that a higher drug release efficiency could be obtained at lower temperatures is reasoned because ICHHPG is more inclined to liquid phase at 4 °C compared to that in 37 °C (Figure 2B), and it suffered less spatial barrier for drug release in the hydrogel matrix. These outcomes suggest that the release efficiency of ICHHPG-loaded CPT is susceptible to the surrounding temperatures, and that is applicable for use on the skin where the temperature is about 32 °C.

To assess the drug release effect of ICHHPG during PTT, the CPT release efficiency under NIR irradiation was further examined. As plotted in Figure 3D, the entrapped CPT can be quickly released upon NIR irradiation and a release ratio of 51.3 ± 8.1% was achieved after 5 min. Given that the temperature of the hydrogel system can be elevated to >70 °C after 2 min of NIR irradiation (Figure 3B), we speculate that the structure of ICHHPG was dramatically changed from hard gel to soft gel (*T*_sg_ of PF127 ~69.8 °C) [35] and thus leading to quick release of CPT. In comparison to the confined drug release effect in the dark as shown in Figure 3C, these outcomes indicate that the release effect of CPT from ICHHPG can be greatly enhanced by NIR irradiation, and that is advantageous for use in the practice.

### 2.5. Rheology and Degradation of ICHHPG In Vitro

**Figure 4:**
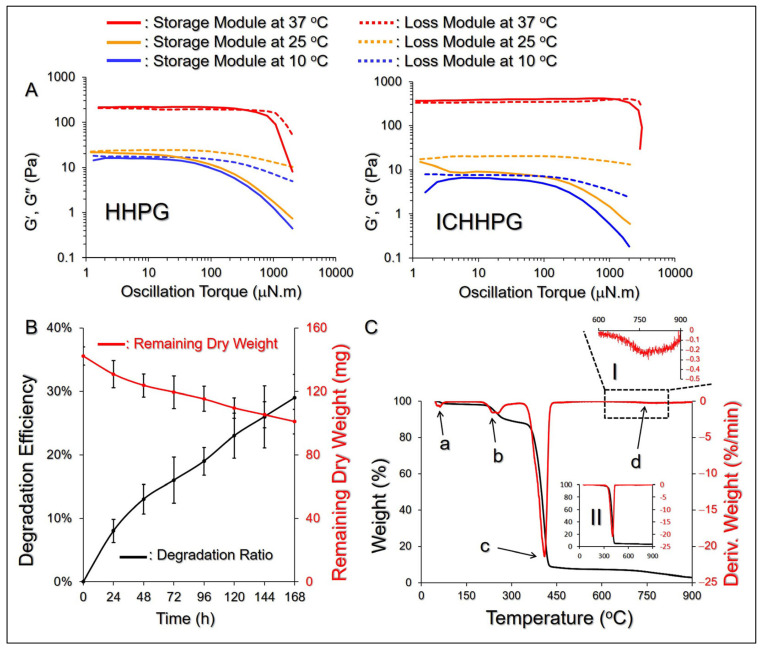
Physicochemical properties of ICHHPG. (**A**) Curves of storage modulus (G′) and loss modulus (G″) vs. oscillation torque for HHPG (left) and ICHHPG (right) under 10, 25, or 37 °C. (**B**) Degradation (black) and dry weight variation (red) profiles of ICHHPG under incubation in 37 °C PBS for 7 days. Values are the mean ± SD (n = 3). (**C**) TGA (black) and DTG (red) curves of ICHHPG heated from 40 to 900 °C with an increasing rate of 10 °C/min under a nitrogen environment. a, b, c, and d points indicate the four peaks on the DTG profile. The inset diagrams show the magnified DTG curve of ICHHPG on 600–900 °C (I) and the whole TGA as well as DTG profiles of HHPG (II).

The mechanical properties of ICHHPG with 40/100 μM of ICG/CPT under different temperatures were assessed by analyzing their rheological behaviors as a function of oscillation torque. As exhibited in Figure 4A, both HHPG and ICHHPG were in a steady liquid state in ≤25 °C, while they shifted to gel phase as the temperature was increased to 37 °C, indicating that the compositions of ICG and CPT may not influence the thermal sensitivity of ICHHPG. Moreover, the characteristics of ICHHPG are that it has a higher viscosity at increasing temperature which is anticipated to enable a stable adhesion on the skin.

The degradation efficiency of ICHHPG was analyzed by measuring the dry weight of the gel under incubation in 37 °C PBS. As plotted in Figure 4B, approximately 102 mg of ICHHPG was retained on the seventh day, which corresponded to *D*_d_ = 29% after the heating process. Such a degradation possibly occurred due to the breakdown of the ester bonds between the acrylate groups and PEO-PPO-PEO block copolymers, leading to decomposition of PF127 micelles upon heating in PBS [36]. Such a mild biodegradability that ICHHPG can still maintain ~70% of gel entity after a week in >30 °C is highly favorable for use on the skin/wound surface.

### 2.6. Thermal Properties of ICHHPG

Figure 4C shows the TGA and DTG profiles of ICHHPG under heating from 40 to 900 °C, where multiple weight-loss peaks representing thermal degradations can be observed in the DTG curve (Figure 4C, points a–d). The first stage of degradation with ~3% weight loss occurred between 50 and 80 °C was likely attributed to loss of adsorbed water. The second degradation, appearing at 160–320 °C with ~6% of weight loss, was highly correlated with the disintegrations of PHMB (decomposition temperature ~190 °C), ICG (*T*_m_ = 235 °C), HPF127 (*T*_b_ of HDI = 255 °C), and HA [37,38]. The third weight loss of ~90% at 320–450 °C was attributed to the decomposition of PF127 [39], while the fourth weight loss of 1% at 600–900 °C likely resulted from further degradations of remaining PF127 and HA.

### 2.7. Cytotoxicity of ICHHPG In Vitro

**Figure 5:**
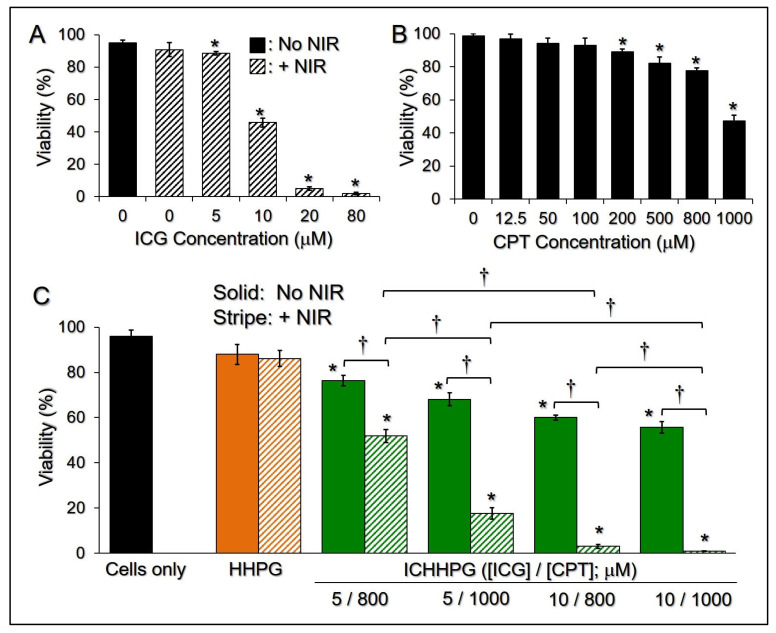
Cytotoxicity of ICHHPG to melanoma in vitro. (**A**,**B**) Viabilities of the B16F10 cells after treatment with various concentrations of ICG + NIR (**A**) or CPT (**B**) for 24 h. Values are the mean ± SD (n = 3). * *p* < 0.05 compared to the group without treatment. (**C**) Viabilities of the B16F10 cells 24 h after treatments with ICHHPG in various conditions. Values are the mean ± SD (n = 3). * *p* < 0.05 compared to the group without treatment (cells only). ^†^ *p* < 0.05.

Figure 5A shows the phototoxicity of ICG with various concentrations to B16F10 cells under NIR exposure. The results show that more than 90% of cells were alive after treatment with NIR alone without ICG, demonstrating that the NIR-induced mild temperature increase (Figure 3B) is not toxic to melanoma. However, ICG + NIR may have provided a dose-dependent cancericidal efficacy and enabled a significant cytotoxicity (<50%) to melanoma when the dose of ICG was elevated to >10 μM (*p* < 0.05). Similar results can be found in the CPT assay. The cell viability dramatically decreased by 51% as the concentration of CPT was increased from 12.5 to 1000 μM (Figure 5B).

We subsequently investigated the photochemotoxicity of ICHHPG with [ICG] = 5 or 10 μM incorporation with 800 or 1000 μM of CPT to B16F10 cells. As plotted in Figure 5C, the viabilities of cells treated with HHPG in the presence and absence of NIR were close (*p* = NS) and those were all similar to the one without hydrogel (*p* = NS for each), indicating that the toxicity of the hydrogel entity is negligible and is independent of NIR exposure. ICHHPG with ≥5/800 μM of ICG/CPT can provide significant anticancer effects compared to ICHHPG without NIR under equal ICG/CPT dosages (*p* < 0.05). With NIR exposure, an increase in either ICG or CPT dosage in ICHHPG dramatically promotes the cytotoxicity of the gel (Figure 5C, *p* < 0.05 for each). Additionally, ICHHPG may provide remarkably enhanced cancericidal efficacy compared to an equal concentration of free ICG (Figure 5A) or CPT (Figure 5B) alone. More than 95% of the cancer cells that can be destroyed by the intra-gel doses of ICG and CPT were elevated to ≥10 μM and ≥800 μM, respectively. These results suggest that both ICG and CPT are critical in ICHHPG-mediated anticancer applications. Although such a high cancericidal efficacy (cell viability < 5%) could be reached by ICG + NIR with [ICG] ≥ 20 μM (Figure 5A), ICG aqueous solution is not suitable for skin cancer post-surgical treatment because it is not easy to retain on the skin surface. Moreover, ICG is photo- and thermally susceptible and easy to be removed from the circulation, which makes it unfavorable for use in the clinic [40]. With the advantages of enhanced ICG stability, considerable hyperthermia efficacy, and effective cancericidal functionality, ICHHPG was anticipated to be a feasible tool for the treatment of skin cancer after surgery.

## 3. Conclusions

In summary, an injectable and thermoresponsive HPF127/HA copolymer crosslinking composite hydrogel encapsulating ICG and CPT, called ICHHPG, was successfully fabricated for photochemotherapy of skin cancer after oncosurgery. ICHHPG can stabilize the entrapped ICG and generate comparable hyperthermia effects with ICG solution upon NIR exposure to perform PTT. The entrapped CPT can be efficiently released upon NIR irradiation and conduct long-term chemotherapy afterward. Taken together, ICHHPG integrating ICG and CPT could provide all-around PTT followed by sustained CPT-derived chemotherapy on the excision site, resulting in successful skin cancer treatment after tumor excision. Such combinational therapeutics may overcome the issues of detrimental side effects and multidrug resistance occurring in most chemotherapies. Given the confirmed cytotoxicity together with its fluidic and thermoresponsive characteristics which are foreseeably favorable for wound coverage, the developed ICHHPG is anticipated to be a feasible tool for use in skin cancer treatment after surgical excision. The in vivo assay is certainly needed and the efforts are currently in progress.

## 4. Materials and Methods

### 4.1. Synthesis of HPF127 Copolymer

About 6 g of PF127 (Sigma-Aldrich, St. Louis, MO, USA) was first heated at 80 °C under vacuum for 30 min. Next, 85 mg of HDI (Sigma-Aldrich, St. Louis, MO, USA) and 50 mg of stannous octoate were added into the melted PF127 and stirred at 80 °C for 30 min. After cooling down to ambient temperature, the mixture was dissolved in 30 mL chloroform followed by dissolution in 200 mL of ethyl ether/petroleum ether (*v*/*v* = 1:1), resulting in precipitation of the synthesized HPF127 out of the solution. The HPF127 was collected by the suction filtration approach and then vacuum-dried at room temperature for 48 h. The synthetic HPF127 was stored at 4 °C and characterized using proton nuclear magnetic resonance (^1^H NMR).

### 4.2. Fabrication and Characterization of ICHHPG

HPF127 was first dissolved in deionized water (10 wt%) through vigorous agitation at 4 °C. Next, HA (MW = 1200 kDa. Sigma-Aldrich, St. Louis, MO, USA) and the desired amounts of ICG (Sigma-Aldrich, St. Louis, MO, USA) and CPT (Sigma-Aldrich, St. Louis, MO, USA) were added to the above HPF127 solution stepwise under stirring where the concentration of HA was 0.5 wt%. The mixture was continuously agitated by 1500 rpm at 4 °C for 30 min to form ICHHPG. The morphologies of surface and inner structure of ICHHPG were detected by SEM.

### 4.3. Evaluation of the Stability and Drug Release Kinetics of ICHHPG

ICHHPG with 40/10 μM of ICG/CPT were placed in 96-well culture plates (200 μL/well) without bubbles inside the gel. After incubation at 4 or 37 °C for 3, 6, 12, 24, and 48 h, ICHHPG was subjected to UV–Vis spectrometry set at λ = 780 nm to analyze the amount of ICG remaining in each sample using Beer–Lambert’s law.

To evaluate the release kinetics of CPT, 5 mL of ICHHPG with 10/100 μM of ICG/CPT was separately added to 20 mL PBS. After incubation at 4 or 37 °C for 3, 6, 12, 24, and 48 h, the supernatant of each group was subjected to UV–Vis spectrometry set at λ = 370 nm to analyze the amount of the released CPT using Beer–Lambert’s law.

To assess how NIR influenced the drug release efficiency from the hydrogel, 100 μL of ICHHPG with 40/100 μM of ICG/CPT and 100 μL of PBS was stepwise placed in 96-well culture plates. After exposed to NIR for 1, 2, 3, 4, and 5 min, 50 μL of the supernatant from each well was subjected to UV–Vis spectrometry set at λ = 370 nm to measure the released CPT as descried above. The temperature of each well under NIR irradiation was concomitantly detected every 60 sec for 5 min. NIR treatment was operated using an 808 nm laser with 6 W/cm^2^ of output intensity.

### 4.4. Evaluation of ICHHPG-Induced Hyperthermia Effects

About 100 μL of ICHHPG containing 2.5, 5, 10, 20, 40, and 80 μM of ICG, in which the CPT concentration was constantly set at 10 μM for each group, was separately added to 100 μL of PBS in 96-well culture plates. Under NIR exposure conducted by using an 808 nm laser with 6 W/cm^2^ of output intensity, the temperature of each group was measured using a digital thermometer every 30 sec for 5 min.

### 4.5. Evaluation of the Rheological and Thermal Properties of ICHHPG

The rheological properties of the HHPG and ICHHPG with 40/100 μM of ICG/CPT were measured using a rheometer (Discovery HR-1, TA Instruments, New Castle, DE, USA) equipped with a temperature controller. The storage (G′) and loss (G″) modules vs. oscillation torque (μN × m) were measured under 37 °C for each sample.

The thermal properties of HHPG and ICHHPG ([ICG]/[CPT] = 40/100 μM) were assessed by thermogravimetric analysis (TGA, PYRIS 1, Perkin Elmer, Shelton, CT, USA) in association with derivative thermogravimetry (DTG). Both lyophilized hydrogel samples were heated from 40 to 900 °C where the increasing rate of temperature was set to 10 °C/min under a nitrogen environment.

### 4.6. Analysis of Degradation of ICHHPG In Vitro

About 7 mL ICHHPG containing 40/100 μM of ICG/CPT was aliquoted into seven tubes. Each ICHHPG sample was added by PBS (ICHHPG:PBS = 1:1 (*v*/*v*)) and incubated at 10, 25, or 37 °C in the dark. One tube of ICHHPG was lyophilized and weighed every 24 h for 7 days. The degradation efficiency (*D*_d_) of ICHHPG was evaluated by *D*_d_ = (*W* − *W*(t))/*W*, where *W* means the original dry weight of ICHHPG before heating, whereas *W*(t) denotes the dry weight of the sample measured at a specific time *t* > 0 after lyophilization.

### 4.7. Cell Culture

Murine melanoma B16F10 cells (ATCC^®^ CRL-6475^™^, ATCC, Rockville, MD, USA) were cultured in Dulbecco’s modified eagle medium (DMEM) supplemented with 10% fetal bovine serum (FBS), 1.5 g/L sodium bicarbonate, and 100 U/mL penicillin/streptomycin at 37 °C with 5% CO_2_ and 100% humidity.

### 4.8. Assessment of Cytotoxicity of ICHHPG In Vitro

The phototoxicity of ICG and chemotoxicity of CPT on melanoma were separately examined before being applied to HHPG. In brief, B16F10 cells in 24-well culture plates (2 × 10^5^ cells/well) were separately treated with 0, 5, 10, 20, and 80 μM of ICG in the presence of 5 min NIR (808 nm, 6 W/cm^2^), or 0, 12.5, 50, 100, 200, 500, 800, and 1000 μM of CPT, and subjected to trypan blue-mediated hemocytometry after incubation at 37 °C for 24 h.

The effects of photochemotherapy of ICHHPG on melanoma were further detected where the dosages of ICG and CPT were determined based on the results of ICG- and CPT-mediated anticancer examinations described above. Briefly, B16F10 cells in 96-well culture plates (1 × 10^5^ cells/well) were separately treated with none (blank), NIR alone, ICHHPG, and ICHHPG + NIR, in which the doses of intra-gel ICG and CPT were set as [ICG]/[CPT] = 0/0 (i.e., HHPG), 5/800, 5/1000, 10/800, and 10/1000 μM. NIR exposure was performed using an 808 nm laser with 6 W/cm^2^ of output intensity for 5 min. All groups were subjected to viability analyses by trypan blue-mediated hemocytometry after incubation at 37 °C for 24 h.

### 4.9. Statistical Analysis

All data were represented as the mean ± standard deviation (SD) with n ≥ 3 unless specified otherwise. Statistical analyses were conducted using the MedCalc software (version 17.2) in which comparisons of one condition between two groups were performed using Student’s *t*-test followed by Dunnett’s post hoc test. *p* < 0.05 was considered statistically significant throughout the study.

## Data Availability

The data presented in this study are available on request from the corresponding author.

## References

[1] Siegel R.L., Giaquinto A.N., Jemal A. (2024). Cancer statistics, 2024. CA Cancer J. Clin..

[2] Fidler M.M., Gupta S., Soerjomataram I., Ferlay J., Steliarova-Foucher E., Bray F. (2017). Cancer incidence and mortality among young adults aged 20-39 years worldwide in 2012: A population-based study. Lancet Oncol..

[3] Cullen J.K., Simmons J.L., Parsons P.G., Boyle G.M. (2020). Topical treatments for skin cancer. Adv. Drug Deliv. Rev..

[4] Quazi S.J., Aslam N., Saleem H., Rahman J., Khan S. (2020). Surgical Margin of Excision in Basal Cell Carcinoma: A Systematic Review of Literature. Cureus.

[5] Golda N., Hruza G. (2023). Mohs Micrographic Surgery. Dermatol. Clin..

[6] Carr S., Smith C., Wernberg J. (2020). Epidemiology and risk factors of melanoma. Surg. Clin..

[7] Wysong A., Higgins S., Blalock T.W., Ricci D., Nichols R., Smith F.L., Kossintseva I. (2019). Defining skin cancer local recurrence. J. Am. Acad. Dermatol..

[8] Holtkamp L.H.J., Lo S.N., Thompson J.F., Spillane A.J., Stretch J.R., Saw R.P.M., Shannon K.F., Nieweg O.E., Hong A.M. (2023). Adjuvant radiotherapy after salvage surgery for melanoma recurrence in a node field following a previous lymph node dissection. J. Surg. Oncol..

[9] Luke J.J., Schwartz G.K. (2013). Chemotherapy in the management of advanced cutaneous malignant melanoma. Clin. Dermatol..

[10] Bhave P., Pallan L., Long G.V., Menzies A.M., Atkinson V., Cohen J.V., Sullivan R.J., Chiarion-Sileni V., Nyakas M., Kahler K. (2021). Melanoma recurrence patterns and management after adjuvant targeted therapy: A multicentre analysis. Br. J. Cancer.

[11] Jazirehi A.R., Lim A., Dinh T. (2016). PD-1 inhibition and treatment of advanced melanoma-role of pembrolizumab. Am. J. Cancer Res..

[12] Worku D.A., Hewitt V. (2020). The role and economics of immunotherapy in solid tumour management. J. Oncol. Pharm. Pract..

[13] Kalal B.S., Upadhya D., Pai V.R. (2017). Chemotherapy Resistance Mechanisms in Advanced Skin Cancer. Oncol. Rev..

[14] Sachet N.F.M., Sajet H.F.M., Al-Owaidi M.M.N., Aljulihawi S.R.M., Al Idreis M.K.J., Al-Gharibawi W.N.A., AlKandali H.A.I., Kamel D.J. (2024). Radiotherapy and Its Associated Side Effects in Breast, Head and neck, Liver, Thyroid, Non-melanoma skin Cancer and Recent Treatment Strategies. Curr. Clin. Med. Educ..

[15] Sánchez-Cid P., Jiménez-Rosado M., Romero A., Pérez-Puyana V. (2022). Novel Trends in Hydrogel Development for Biomedical Applications: A Review. Polymers.

[16] Khan S., Akhtar N., Minhas M.U., Badshah S.F. (2019). pH/Thermo-Dual Responsive Tunable In Situ Cross-Linkable Depot Injectable Hydrogels Based on Poly(NIsopropylacrylamide)/Carboxymethyl Chitosan with Potential of Controlled Localized and Systemic Drug Delivery. AAPS PharmSciTech.

[17] He J., Zhang Z., Yang Y., Ren F., Li J., Zhu S., Ma F., Wu R., Lv Y., He G. (2021). Injectable Self-Healing Adhesive pH-Responsive Hydrogels Accelerate Gastric Hemostasis and Wound Healing. Nano-Micro Lett..

[18] PJ R.J., Oluwafemi O.S., Thomas S., Oyedeji A.O. (2022). Recent advances in drug delivery nanocarriers incorporated in temperature-sensitive Pluronic F-127–A critical review. J. Drug Deliv. Sci. Technol..

[19] Shamma R.N., Sayed R.H., Madry H., El Sayed N.S., Cucchiarini M. (2022). Triblock Copolymer Bioinks in Hydrogel Three-Dimensional Printing for Regenerative Medicine: A Focus on Pluronic F127. Tissue Eng. Part B Rev..

[20] Lippens E., Swennen I., Gironès J., Declercq H., Vertenten G., Vlaminck L., Gasthuys F., Schacht E., Cornelissen R. (2013). Cell survival and proliferation after encapsulation in a chemically modified Pluronic(R) F127 hydrogel. J. Biomater. Appl..

[21] Peng S., Lin J.Y., Cheng M.H., Wu C.W., Chu I.M. (2016). A cell-compatible PEO-PPO-PEO (Pluronic^®^)-based hydrogel stabilized through secondary structures. Mater. Sci. Eng. C Mater. Biol. Appl..

[22] Lane D. (2006). Designer combination therapy for cancer. Nat. Biotechnol..

[23] Xie Z., Fan T., An J., Choi W., Duo Y., Ge Y., Zhang B., Nie G., Xie N., Zheng T. (2020). Emerging Combination Strategies with Phototherapy in Cancer Nanomedicine. Chem. Soc. Rev..

[24] Raza A., Hayat U., Rasheed T., Bilal M., Iqbal H.M.N. (2019). “Smart” Materials-Based Near-Infrared Light-Responsive Drug Delivery Systems for Cancer Treatment: A Review. J. Mater. Res. Technol..

[25] Li J., Wang S., Fontana F., Tapeinos C., Shahbazi M.A., Han H., Santos H.A. (2022). Nanoparticles-based phototherapy systems for cancer treatment: Current status and clinical potential. Bioact. Mater..

[26] Lee Y.H., Pham U.N.T. (2023). Engineered indocyanine green and PD-L1 inhibitors co-loaded perfluorochemical double-layered nanodroplets offer effective photoimmunotherapy against colorectal cancer. Chem. Eng. J..

[27] Jiang Z., Li J., Chen S., Guo Q., Jing Z., Huang B., Pan Y., Wang L., Hu Y. (2020). Zoledronate and SPIO dual-targeting nanoparticles loaded with ICG for photothermal therapy of breast cancer tibial metastasis. Sci. Rep..

[28] Ma R., Alifu N., Du Z., Chen S., Heng Y., Wang J., Zhu L., Ma C., Zhang X. (2021). Indocyanine Green-Based Theranostic Nanoplatform for NIR Fluorescence Image-Guided Chemo/Photothermal Therapy of Cervical Cancer. Int. J. Nanomed..

[29] Saxena V., Sadoqi M., Shao J. (2003). Degradation Kinetics of Indocyanine Green in Aqueous Solution. J. Pharm. Sci..

[30] Mindt S., Karampinis I., John M., Neumaier M., Nowak K. (2018). Stability and Degradation of Indocyanine Green in Plasma, Aqueous Solution and Whole Blood. Photochem. Photobiol. Sci..

[31] Veloso A., Biewen B., Paulsen M.T., Berg N., Carmo de Andrade Lima L., Prasad J., Bedi K., Magnuson B., Wilson T.E., Ljungman M. (2013). Genome-wide transcriptional effects of the anti-cancer agent camptothecin. PLoS ONE.

[32] Huang G., Huang H. (2018). Application of hyaluronic acid as carriers in drug delivery. Drug Deliv..

[33] Russo E., Villa C. (2019). Poloxamer Hydrogels for Biomedical Applications. Pharmaceutics.

[34] Zarrintaj P., Ramsey J.D., Samadi A., Atoufi Z., Yazdi M.K., Ganjali M.R., Amirabad L.M., Zangene E., Farokhi M., Formela K. (2020). Poloxamer: A versatile tri-block copolymer for biomedical applications. Acta Biomater..

[35] Russo G., Delpiano G.R., Carucci C., Grosso M., Dessì C., Söderman O., Lindman B., Monduzzi M., Salis A. (2024). Tuning Pluronic F127 phase transitions by adding physiological amounts of salts: A rheology, SAXS, and NMR investigation. Eur. Polym. J..

[36] Kim M.R., Park T.G. (2002). Temperature-responsive and degradable hyaluronic acid/Pluronic composite hydrogels for controlled release of human growth hormone. J. Control. Release.

[37] De Paula G.F., Netto G.I., Mattoso L.H.C. (2011). Physical and Chemical Characterization of Poly(hexamethylene biguanide) Hydrochloride. Polymers.

[38] Lopez K.M., Ravula S., Pérez R.L., Ayala C.E., Losso J.N., Janes M.E., Warner I.M. (2020). Hyaluronic Acid-Cellulose Composites as Patches for Minimizing Bacterial Infections. ACS Omega.

[39] Nguyen D.T., Dinh V.T., Dang L.H., Nguyen D.N., Giang B.L., Nguyen C.T., Nguyen T.B.T., Thu L.V., Tran N.Q. (2019). Dual Interactions of Amphiphilic Gelatin Copolymer and Nanocurcumin Improving the Delivery Efficiency of the Nanogels. Polymers.

[40] Mordon S., Devoisselle J.M., Soulie-Begu S., Desmettre T. (1998). Indocyanine green: Physicochemical factors affecting its fluorescence in vivo. Microvasc. Res..

